# Swallowable Wireless Capsule Endoscopy: Progress and Technical Challenges

**DOI:** 10.1155/2012/841691

**Published:** 2011-12-27

**Authors:** Guobing Pan, Litong Wang

**Affiliations:** ^1^Key Laboratory of E&M, Zhejiang University of Technology, Ministry of Education, Hangzhou 310032, China; ^2^College of Science, Zhejiang University of Technology, Hangzhou 310032, China

## Abstract

Wireless capsule endoscopy (WCE) offers a feasible noninvasive way to detect the whole gastrointestinal (GI) tract and revolutionizes the diagnosis technology. However, compared with wired endoscopies, the limited working time, the low frame rate, and the low image resolution limit the wider application. The progress of this new technology is reviewed in this paper, and the evolution tendencies are analyzed to be high image resolution, high frame rate, and long working time. Unfortunately, the power supply of capsule endoscope (CE) is the bottleneck. Wireless power transmission (WPT) is the promising solution to this problem, but is also the technical challenge. Active CE is another tendency and will be the next geneion of the WCE. Nevertheless, it will not come true shortly, unless the practical locomotion mechanism of the active CE in GI tract is achieved. The locomotion mechanism is the other technical challenge, besides the challenge of WPT. The progress about the WPT and the active capsule technology is reviewed.

## 1. Introduction

Most diseases such as bleeding, ulcer, and tumor can be cured or controlled in their early stages, or they will deteriorate into cancer or some other vital diseases [1]. Diagnosing these diseases in their early stages is of great importance, but it is not easy. Many indirect technologies have been developed to detect GI tract diseases such as angiography, ultrasonography, X-radiography (including CT), and scintigraphy. Unfortunately, they were reported to have low diagnostic yields even for bleeding detection [2, 3] or be rarely helpful unless the bleeding is active severely [4].

The best way to detect GI diseases and uncover the inner works is directly viewing the GI tract, so the endoscopy is a direct and effective diagnostic technology [5]. The invention of wired endoscopy made it possible to view the entire stomach, the upper small intestine, and colon. Because of the ability of allowing clinicians to directly view the GI tract, endoscopy has become the standard method and the criteria for diagnosing GI diseases in clinic. However, limited by physical reasons, the traditional invasive wired endoscopy cannot examine the whole GI tract, leaving the small intestine as a dead zone. They are inconvenient and cause intense pain for patients. Furthermore, they can increase the risk of intestine perforation and the chances of cross-contamination.

In 2000, a new kind of endoscopy, wireless capsule endoscopy (WCE), was reported by Given Imaging Company. The WCE system constitutes of four parts including the capsule endoscope (CE), the data receiving box, the working station, and the application software. The CE is 11 mm in diameter and 30 mm in length, which is small enough to be swallowed by patients easily. During its travelling through the GI tract with the peristalsis, the CE images the GI tract. The images are transmitted wirelessly outside of patient's body and received by the receiving box which is tied to patient's waist. The CE is powered by a cell battery, which can keep working continuously up to 6 hours [6, 7]. The first clinical trial was carried out in 2001 [8]. The usage of WCE technology is convenient and safe, and the entire GI tract is examined without dead zones. Such endoscopy is a realistic alternative to traditional invasive endoscopy and revolutionizes the methods of diagnosing the GI tract diseases.

The WCE has bloomed into an important diagnosis technology in clinic based on its assets. It has irreplaceable effects especially on the small intestine diagnosis and is mainly used to diagnose bleeding, ulcer, tumour, and others. Many kinds of WCE have been developed so far, and several commercial WCE products are available in the market. However, the limited working time, the low frame rate, and the low image resolution of WCE limit the wider application. Therefore, the tendencies of this novel technology are toward the high frame rate, high image resolution, and long working time. In the mean time, the CE locomotion now is passive, and so its position cannot be controlled, which is the main drawback of the WCE technology. The active CE, namely the capsule robot, is the next important tendency of WCE. Aiming at the tendencies, the research work that scientists are engaged in can be classified into four technologies: the technology of CE, the technology of image processing, the technology of wireless power transmission (WPT), and the technology of locomotion mechanism of the active CE. The image processing technology can be separated from the technology of WCE, and so it will not be introduced here. In this paper, the progress of the WCE technology will be summarized. The tendencies of WCE evolution and the relevant technical challenges will be analyzed. Finally, the progress of WPT and locomotion mechanisms of active CE is reviewed.

## 2. Overview of Swallowable Capsule Endoscopy

### 2.1. Early Wireless Swallowable Capsule and Wired Endoscopy

The main physiological parameters of GI tract, which concern physicians, are pH value, temperature, pressure, oxygen, and conductivity. These physiological parameters change slightly and regularly within a certain value range. Therefore, the information of physiological parameters can reflect the pathologic changes and consequently is helpful for early diagnosis.

The first swallowable parameter capsule was developed in 1950s to measure the pressure information in the small intestine of patients with dysentery [9–11]. After that, the temperature parameter capsule [12] and the pH parameter capsule [13] were developed. However, limited by the electronic technology, these parameters capsules had low integrated level, bad performance, and prohibitive costs. Therefore, it is hardly for them to be applied in clinic.

Germany Heidelberg Medical Inc. developed the first commercial pH parameter capsule detection system. The capsule, which is 7.1 mm in diameter and 15.4 mm in length, can keep working in GI tract for 6 hours. This device is used to diagnose the gastric hyperacidity, the anachlorhydria [14]. In 2002, American Medtronic Company released another wireless pH capsule system, which is named Bravo. This system is mainly used to diagnose the gastric acid reflux disease [15]. A new multiparameter capsule system named SmartPill was developed by American SmartPill Company. SmartPill has the capability of measuring the pressure, temperature, and pH value of GI tract at one time. Now, this product has gotten the FDA (Food and Drug Administration) license.

The physiological parameters capsule measurement devices are helpful for early diagnosis and prophylactic treatment. However, these kinds of swallowable capsule are indirect diagnosis technology with low reliability. The best way to diagnose GI diseases is directly viewing the GI tract wall. The invention of the wired endoscopy made visualization of the entire stomach, upper small intestine, and colon possible. Since 1950, many kinds of wired endoscope have been invented and used in the clinical field, such as gastroscope, esophagoduodenoscope, and colonoscope. The development history of wired endoscopy can be divided into four stages: rigid-wire endoscopy, semiflexible lens endoscopy, fiber-optic endoscopy, and digital endoscopy. Now, the fiber-optic endoscopy and digital endoscopy are still the main diagnosis technologies of GI diseases.

The key part of the fibre-optic endoscope is the fibre bundle, which is used to carry images out. The fibre bundle is composed of tens of thousands of very fine glass fibers, and the fine glass fiber can carry the reflected image out. From 1963, Japanese companies began to make the fibre-optic gastroscope product, and the gastroscope was improved to be more practicable. Moreover, the biopsies devices were embedded in this fibre-optic endoscope, so the functions were widely extended. The digital endoscope tows flexible and long cables, which carry power, video signal, and lighting. On the top end of these cables, a microcamera is assembled. During the examination course, the digital wired endoscope is pushed into the GI tract, with these cables. Lighting source through the fibre-optic bundles lights the GI tract and the microcamera obtains the images for inspection. Compared with the earlier wired endoscopes, digital endoscope can get more clear images with higher image resolution. Moreover, the image can be stored and processed with computer. This kind of wire endoscope is now popular in clinic. Now, Welch Allyn Company, Olympus and Fujinon are successful vendors of digital wired endoscopes.

### 2.2. WCE and the Commercial Products

Wired endoscopy provides a practical technology for physicians to view the GI tract. However, limited by physical reasons, these traditional invasive endoscopies cannot be pushed through the entire GI tract, leaving the small intestine as a dead zone. They are inconvenient and cause intense pain. Further, they can increase the risk of intestine perforation and the cross-contamination.

**Figure 1:**
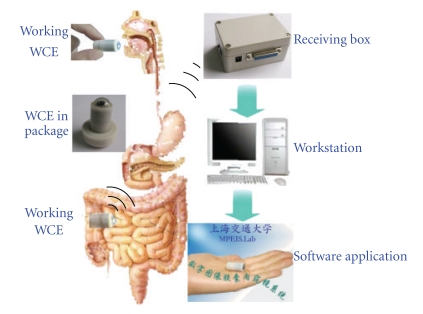
A typical WCE system.

The technological breakthroughs, including semiconductors, integrated circuits, illumination, and the greater understanding of human physiology, made possible development of a new type of endoscopy—wireless capsule endoscopy (WCE). A typical WCE system contains four parts: capsule endoscope (CE), receiving box, image working station, and software application, as shown in Figure 1 [16]. The CE is an electronic microsystem, which can be ingested to image the GI tract and transmit the images outside of the patient's body.

**Figure 2:**
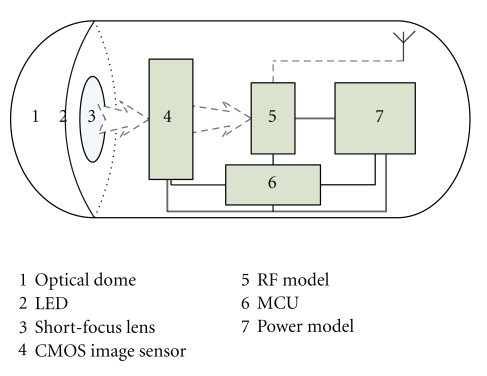
The architecture of CE.

After reporting the first WCE system in 2000, the Given Image Company released the first commercial WCE product system M2A in Yoqneam, Israel [6]. The CE of the system is 11 mm in diameter and 26 mm in length, which is small enough to be swallowed by the patient. The CE is composed of 7 main models including optical dome, short-focus lens, CMOS (complementary metal oxide semiconductor) image sensor, RF (radio frequency) transmitter, MCU (microcontrol unit), LED (light emission diode) lighting, and cell battery, as shown in Figure 2. After the CE is swallowed by patients, it will go through the entire GI tract with the natural peristalsis. During this course, the optical dome can plump up the intestine wall without requiring air inflation. In the mean time, the micro CMOS image sensor images the GI tract, and the RF model transmits the images outside of patient's body at the frame rate of 2 f/s (frames per second). The images are received by the receiving box outside and showed in PC workstation. After the examination, the CE is vented out naturally. The CE is powered by a cell battery, which allows more than 6 hours of continuous working. The first human clinical trial was carried out in 2001, and the FDA was issued to M2A in August 2001 [7, 8, 17]. Such WCE offers a convenient examination with minimal preparation and immediate recovery. It is a realistic alternative to traditional wired invasive endoscopy and revolutionizes the methods of inspecting the GI tract.

**Figure 3:**
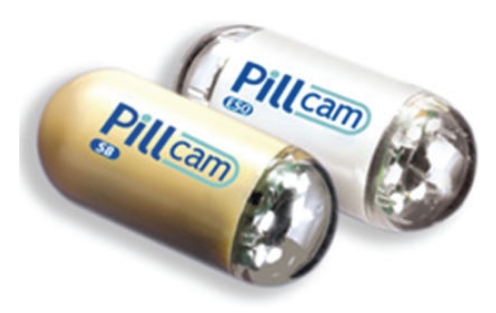
PillCam SB and PillCam ESO.

The name of M2A was changed to PillCam (means Pill and Camera) later. In 2005, Given Imaging developed two distinct WCE systems: PillCam ESO specially for the esophagus [18] and PillCam SB specially for the small intestine [19], as shown in Figure 3. The two kinds of CEs have the same architecture and the same working principle to M2A. PillCam SB is 11 × 26 mm of outline and weights less than 4 g, and its continuous working time is 7 ± 1 hours. PillCam ESO has the same dimensions to PillCam SB and is equipped with miniature cameras on both ends. During the five-minute examination of passing down the esophagus, PillCam ESO has the capability of capturing 18 images per second, and its continuous working time is 20 ± 5 minutes. PillCam SB and PillCam ESO are replaced now by the second generations, which integrate advanced optics and automatic light control and so can provide optimal image quality and illumination.

PillCam COLON is another product of Given Imaging, which specially aims at visualization of the colon mucosa and detecting polyps. After FDA Rejected PillCam Colon application in USA in 2008, Given Imaging developed the second-generation PillCam COLON 2 and received a CE Mark in 2009 and was commercially available in Europe in 2010. PillCam COLON 2 is equipped with two image sensors on both ends and provides a near 360° view of the colon. It measures 11 × 31 mm. The most outstanding character of PillCam Colon 2 is the bidirectional communication between the CE and the data recorder. Therefore, the image capture rate can be adjusted in real time from 4 f/s up to 35 f/s to maximize colon tissue coverage and can keep working approximately for 10 hours.

Beside Given Imaging, Olympus Optical Company is another main vendor of WCE. Olympus received the first major patents in 1981 and released its WCE named EndoCapsule in Hamburg, Germany in 2005 [20], which measures 11 × 26 mm. Unlike M2A, this CE is equipped with six white LEDs and a supersensitive CCD (charge-coupled device) image sensor, so it is reported to generate high-resolution images, and the working time in the GI tract can be 8 hours. The latest capsule endoscope from Olympus was reported to have the ability to activate and deactivate itself, but this kind of capsule endoscope is not freely available on the market.

Since the initial launch of M2A, several kinds of WCE were developed, and all of them operate on the same principle. OMOM was released by Chinese Chongqing Jingshan Company and received its sFDA in March 2005. The CE size is 11 × 25.4 mm with a field of view of 140°. The frame rate is 2 f/s, and the longest operation time is 6~8 hours. Now, it is widely used in Chinese hospital [21, 22]. MicroCam was released by IntroMedic Company in Seoul, Korea, in April 2007. The CE size is 11 × 24 mm with a field of view of 150°. The highest frame rate can be 3 f/s, and the longest operation time reaches more than 11 hours [23, 24]. In 2001, RF System Lab released an optimal CE model of Norika. In the design model, Norika is 9 × 23 mm of outline and is equipped with CCD image sensor with the frame rate of 30 f/s. Its four illumination LEDs have different light wavelengths, which can generate simulative 3D images. The focus of the camera lens can be adjusted to obtain more clear images. The most salient characteristic of Norika is the *in vivo* drug delivery and sample extraction. To tackle the power requirement, Norika is powered wirelessly [25].

### 2.3. Advantage and Limitation

WCE is applied more and more not only because of its convenience and being pain-free, but also because of its clinical effects. Comparing WCE with push endoscopy, the appearances of GI tract viewed with WCE were generally similar to those of push endoscopy, and WCE was significantly superior to push endoscopy in the identification of bleeding sources in the small intestine [26]. WCE was also significantly superior to push endoscopy when the total diagnostic yield was analyzed including other GI tract abnormalities as well as bleeding [2, 4, 5, 27–30]. Over all, WCE is an effective means of diagnosing GI tract with high diagnostic yield and reliability. In addition, it is the only imaging method that can provide color images of the lower small intestine painlessly. Furthermore, not considering the cost, patients prefer WCE to traditional push endoscopy.

Nevertheless, there are limitations to WCE. Firstly, the picture quality is not as good as the best quality of flexible wired video endoscopy. The frame rate with the capsule is lower (2–18 v 25 f/s). The image resolution of 256 × 256 is not satisfied, and the light intensity cannot yet respond to altering requirements. Secondly, most of these existing CEs are powered by cell batteries, because of the limited power supply, and few of these CEs can keep working for more than 8 hours. Nevertheless, for a patient with GI tract disease, it needs greatly more than 8 h for diagnosing; otherwise, the small intestine is still not examined when the cell battery runs out of power. Finally, the move of CE is passive, and its location cannot be controlled, so lesions cannot be repeatedly examined. Therefore, WCE is still an immature technology, and technical improvements need to be encouraged.

## 3. The Progress Tendency and Technical Challenges

The WCE technology has been applied in clinic successfully, especially in the small intestine diagnosis. However, compared with wired endoscopy, the limited working time less than 8 h, the low frame rate of 2 f/s, and the low image resolution of 256 × 256 limit the wider application. Not only the working time is absolutely limited by the power supply of the CE, but the image resolution and the frame rate are also limited by the power supply. The parts, which consume most power, include the image sensor, the MCU, and the RF module. Now Given Imaging Company and Chinese Hitron Company in Hangzhou do not use the general IC chips to design the CE. They customized an exclusive IC chip that integrates the MCU module and RF module. Thus, the bulk of the IC chip is decreased significantly, the power consumer of the CE is decreased dramatically, and consequently the working time of the CE is extended to more than 15 hours. However, it is not enough. If improving the image resolution and the frame rate, the power consumer will increase significantly, and the working time will be decreased dramatically. Therefore, the power supply limitation is still the bottleneck for mature WCE system.

One tendency of this novel technology is towards the high frame rate and high-resolution images. The living GI organs are constantly moving, and so the frame rate of 2 f/s is obviously not enough for diagnosing the details of GI tract organs. The ideal frame rate of WCE is as high as video WCE. Now, some video WCE systems have been developed, and the CE can transmit GI tract images with the frame rate of 30 f/s in NTSC video format [31]. On the other side, the image resolution of 256 × 256 is not satisfied compared with wired endoscopy, and the image resolution cannot be too higher any more. However, the high frame rate and high image resolution consequently bring much more power needed which is beyond any cell battery can supply. In order to reduce the image data for the high image resolution and high frame rate, some new image compression algorithms were developed for video CE [32, 33]. However, the complex compression algorithm is not suitable for the tiny and low-power CE, and the effects are limited. Wireless power transmission (WPT) is the right solution for this problem, but it is not easy, and so it is one big technical challenge.

The wireless electric power transmission system is based on electromagnetic induction. Such WPT system has been successfully applied in TET (transcutaneous energy transmission) systems to provide power for some implanted artificial organs [34–36]. But the existing technology for TET systems is not suitable for WCE. In TET systems, the transmitting coils (TCs) and receiving coils (RC) are placed as nearly as possible, and the distance between them is not more than 20 mm. However, the TC and RC in WCE system are placed out and in the body, and the distance between them is 50 mm~150 mm. The longer the distance is, the weaker the transmitting electromagnetic field becomes, so the electric power generated in RC is very limited. Additionally, the RC and TC in TET systems are placed immovably, so their relative position and the RC's orientation are fixed, while the position and orientation of the RC in WCE system is changing constantly with the advancing of the CE in the GI tract. Therefore, the mismatching of the moving RC and the TC will reduce the induced electric power. Theoretically more powerful magnetic field can be generated by increasing the electric power of the TC and so increase the electric power in RC. While this device is used in human body, and increasing highly the magnetic field will do harm to human body.

Another tendency of WCE progress is the active CE, namely, the capsule robot. Now in the existing WCE system, after being swallowed, the CE travels ahead with the GI tract natural peristalsis, and so the CE locomotion is passive. Consequently, the position cannot be controlled, which is the main drawback of the WCE technology. Because of the passive locomotion of the CE, the focus of disease cannot be viewed repeatedly. Moreover, the passive locomotion is the bottleneck of the novel CE with various functions including biopsy and drug delivery. The active CE will be the next generation of the passive CE. Nevertheless, the locomotion of the active CE in GI tract is another big challenge besides of the power supply difficulty. The GI tract is a very difficult environment for active locomotion. The tissue is normally soft and viscoelastic, and it is very difficult for the moving active CE not to harm the tissue. The GI morphology varies along the GI tract, and consequently, the direction of movement will be up and down, besides ahead and back. Moreover, the surface is slippery and varies in thickness and viscosity along the different tracts. Therefore, the locomotion mechanism is the other technical challenge. Some researchers embedded a magnetic actuator in the CE and used a strong magnetic field outside to control the CE to move [37–39]. This kind of self-propelled CE needs a complex actuating system and is hard to use in clinic. Furthermore, the examination is limited and professional, which conflicts with the convenience of WCE. Therefore, it is just the transitional one, not a real active CE.

The high frame rate and high image resolution are the tendency of the CE progress, but the WPT is the big technical challenge. The active CE is another progress tendency, and the active CE can easily integrate new functions such as biopsy and drug delivery. However, beside the WPT, the locomotion mechanism of CE in GI tract is another big technical challenge.

## 4. The Progress of WPT and Locomotion Mechanism

**Figure 4:**
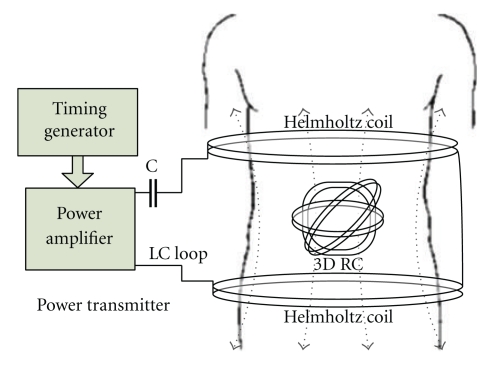
The illustration of the power supply system.

A typical wireless power transmission system is shown in Figure 4, which is the power source of a video WCE system [31]. No cell battery is equipped in the video CE. The wireless power supply system contains the outside power transmission device and the inner power receiving subsystem. The outside power transmission device is composed of a timing generator, a circuit driver, and a Helmholtz transmitting coil (TC). The signal generated by the timing generator is amplified in the circuit driver and used to drive the Helmholtz TC, which generates uniform electromagnetic field in the human body. The power-receiving subsystem is embedded in the CE and is composed of the rectifier/voltage regulator circuit and a three-dimensional orthogonal receiving coil (RC) with a ferrite core. In the changing electromagnetic field, the 3D orthogonal RC generates inducing current, just like the work of a transformer. The induced electric current is rectified and voltage regulated to power the CE.

The 3 coils perpendicular to each other enable the RC generate enough power at any orientation. The ferrite cube core, which can improve the transmission efficiency significantly [40], is made of Mn-Zn material with high initial permeability and low loss factor. The TC is a pair of Helmholtz coils, which consists of two identical circular magnetic coils that are placed symmetrically and separated by the distance *h* equal to the radius *R* of the coil. Each coil carries equal electrical current flowing in the same direction. With the cooperation between Helmholtz TC and the 3D RC, the wireless power supply system can produce stable power for the CE, no matter where and what orientation the CE is [41]. Supplied with the wireless power, the CE has the abilities of getting images of the GI tract with the image resolution of 320 × 240, and transmitting the video outside of body with the frame rate of 30 f/s.

The most salient character of the WPT system for CE is the mismatching of the moving RC and the TC. Soma Mani presented a detailed theoretical analysis of misalignment effects in WPT systems, including lateral and angular misalignments [42]. Kopparthi developed an experiment WPT system. The power-receiving device was fixed in the TC, and highest power gotten in the RC is 1 W when the device was at best condition [43]. However, in this WPT system, the position and the orientation of the CE changes, and the inducted current disappears because of the mismatching between the RC and the TC.

**Figure 5:**
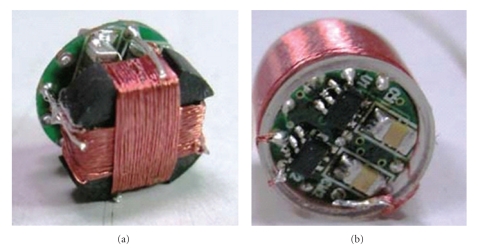
3D receiving coils.

Ryu et al. in Hallym University and his team focused on the impact of the RC coil turns and the load impedance matching to the received power in RC [41, 44]. Aiming to power the CE wirelessly, they designed firstly the 3D RC, which can reduce the impact of the posture of RC. The power receiving system, as shown in Figure 5, can generate 300 mW electric power regardless the orientation of the RC in the magnetic field. Lenaerts et al. and Carta et al. in Belgium Katholieke Universiteit Leuven found that the ferrite core could improve the transmission efficiency significantly. They developed a wireless power transmission system to power the active CE, and the 3D RC was reeled around a cube ferrite core. Using this system, 330 mW electric power was successfully got in the RC [45–50].

Xin et al. and Guan-ying et al. studied the wireless power transmission efficiency by the theory of coupling coefficients, and a WPT system was developed to power the video CE. They did some study work about the safety of the wireless electromagnetic wave [51, 52].

Even many creative WPT systems were developed, and some achievements have been gotten, but it is still a big technical challenger for WCE. The power generated in the RC and the transmission efficiency rise steadily with the frequency and the intensity of the electromagnetic wave. The bigger the electromagnetic intensity and the higher the frequency is, the more power the RC can generate. However, as a kind of conductor, the biological tissue of body also absorbs the electromagnetic wave, and the absorption has the same regular law to that of the RC. Consequently, the bigger the electromagnetic intensity and the higher the frequency is, the greater damage the human body will get [53]. In conclusion, the transmission efficiency still conflict with the safety, but few achievements about the safety study have been gotten. Therefore, more creative WPT and more safety studies must be achieved before the WPT applies in clinic.

The locomotion of the existing CE is passive. Therefore, the movement of the CE cannot be controlled, and the disease focus cannot be viewed repeatedly. Moreover, the functions of biopsy, drug delivery, and other microsurgery cannot be integrated in the CE. The active capsule, namely the capsule robot, is a new prospective instrument expected to substitute for the traditional endoscope and the capsule endoscope. The active capsule can go forward, backward, or stand under the control.

Several prototypes have been developed by researchers in the last 20 years. Dario and his team in Italy CRIM (Center for Research In Microengineering) lab developed an inchworm-like robotic colonoscope, which is driven by some pneumatic actuators that must carry an air tube while moving in the GI tract [54–60]. The active robot colonoscope is 22 mm in diameter and 153 mm in length. There are many fine holes in the front and hinder sections. The fine holes can absorb the wall of GI tract by the air pumping of pneumatic actuators, and so the section can be controlled to stop. The middle section pushes the front section and draws the hinder section in phase, and so the active robotic colonoscope moves ahead. Unfortunately, this mechanism applies abnormal pressure on the GI inner wall for enough friction force, which may injure the thin and vulnerable human tissue. In 2004, this team developed another active endoscope which is driven by shape memory alloy (SMA) actuator [61, 62]. The robot capsule has eight agile bioinspired legs, which are made of SMA. The head of the active capsule and the microimage sensor are assembled in an expanded transparent device, which can extent the view filed. However, the locomotion mechanism cannot be applied in clinic because the active endoscope with a piezoelectric linear actuator, or a micromotor, must tow an electric cable. The cable or an air tube not only decreases the robot's flexibility, but also prevents the robot from entering into the deeper place of the GI tract.

Kim et al. of Korea Institute of Science and Technology developed another earthworm-like active CE using microbrushless Dc motors, ionic polymer metal composite actuator, and SMA. In the prototypes, four spring-type SMA actuators are selected to be microactuators for active CE, and four biomimetic clampers are selected to be the stopping devices. It is 13 mm in diameter and 33 mm in total length, with a hollow space of 7.6 mm in diameter to house other parts of CE [63, 64]. However, these kinds of SMA actuators need to be heated and cooled frequently. In a closed environment of the GI, the actuator's cooling speed is very low, so the robot advances at a very low speed.

 Another novel paddling-based locomotive mechanism was proposed [65–67], which was originated from paddling a canoe. The synchronized multiple legs of the active CE are just like the paddles of a canoe. A linear actuator, which is composed of a micromotor and a lead screw, is selected to be microactuators. Two mobile cylinders inside of the capsule are the stopping devices. By the kinematic relation between the legs and the mobile cylinders, the microrobot can move forward in GI tract. There are other novel locomotive mechanisms of earthworm-like and inchworm-like proposed in [68–72].

**Figure 6:**
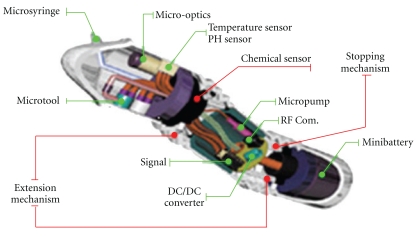
The concept prototype of the active medical robot.

Several kinds of novel locomotion mechanism have been proposed, and some active capsule endoscope prototypes have been developed. However, none of them can move smoothly with steady pace in GI tract so far, and consequently, there is no locomotion mechanism that can be applied in clinic as shortly as the alternative for WCE. Therefore, it is necessary to explore a safe and reliable locomotion mechanism first before the active CE is practical in clinical application. The WCE system with high resolution and high frame rate is the feasible noninvasive diagnoses technology in clinic so far. Nevertheless, the active medical robot with multifunctions is the optimal diagnostic and therapeutic instrument of GI diseases. The function of this kind of active medical robot is not only limited to the endoscopy, but also has the functions of biopsy, parameter sensors, drug delivery, microsurgery, and so on. With the advance of MEMS (microelectromechanical systems), it will probably become a reality. A concept prototype of this kind of medical robot, as shown in Figure 6, is proposed by Korean IMC (Intelligent Microsystems Center). It is only 10 mm in diameter and 20 mm in length. The concept prototype integrates the micro-optical images module, the physiological parameter sensors, the micromechanical arm and pump for biopsy, drug delivery and microsurgery, and the locomotion parts. It has the ability of advancing forward and backward, orientating, stopping, and anchoring itself onto the GI tract wall under the control outside. It is equipped with a micromanipulator arm that is able to perform therapeutic procedures like taking biopsy, microsurgery, and injection.

## 5. Conclusion

WCE offers a feasible noninvasive way to detect the entire GI tract and brings about a revolution in the diagnosis technology of GI diseases. Unfortunately, compared with wired endoscopies, the limited working time, the low frame rate, and the low image resolution limit the wider application. Therefore, the tendencies of the WCE progress are the WCE with high image resolution, high frame rate, and long working time. However, the power supply of CE is the bottleneck of this tendency. WPT is the promising solution to this problem, but is also the technical challenge. Many achievements about the WPT technology have been gotten, but the research efforts are still needed on the safety and transmission efficiency.

Active CE is another tendency and will be the next generation of the WCE, but not shortly. The medical robot, which integrates the multifunctions of the endoscopy, the biopsy, the drug delivery, and the microsurgery, is the optimal diagnostic and therapeutic instrument of GI diseases. However, the locomotion mechanism of it in GI tract is the other technical challenge, besides the challenge of WPT. Some novel and imaginative locomotion mechanisms are proposed, but none of them is practicable to be applied in clinic. Therefore, the novel and practicable locomotion mechanism of the active CE in GI tract, as well as the WPT technology, must be achieved before the clinical application, which is the premise of the active CE or the medical robot.
